# The prognosis for individuals on disability retirement An 18-year mortality follow-up study of 6887 men and women sampled from the general population

**DOI:** 10.1186/1471-2458-6-103

**Published:** 2006-04-22

**Authors:** Thorne Wallman, Hans Wedel, Saga Johansson, Annika Rosengren, Henry Eriksson, Lennart Welin, Kurt Svärdsudd

**Affiliations:** 1 Department of Public Health and Caring Sciences, Family Medicine and Clinical Epidemiology Section, University Hospital, Uppsala, Sweden; 2 Centre for Research & Development in Primary Care (AmC), Eskilstuna, Sweden; 3 Nordic School of Public Health, Gothenburg, Sweden; 4 Department of Medicine, Sahlgrenska University Hospital/Östra, Gothenburg, Sweden; 5 Department of Epidemiology, AstraZeneca R&D Mölndal, Sweden

## Abstract

**Background:**

Several studies have shown a markedly higher mortality rate among disability pensioners than among non-retired. Since most disability pensions are granted because of non-fatal diseases the reason for the increased mortality therefore remains largely unknown. The aim of this study was to evaluate potential explanatory factors.

**Methods:**

Data from five longitudinal cohort studies in Sweden, including 6,887 men and women less than 65 years old at baseline were linked to disability pension data, hospital admission data, and mortality data from 1971 until 2001. Mortality odds ratios were analyzed with Poisson regression and Cox's proportional hazards regression models.

**Results:**

1,683 (24.4%) subjects had a disability pension at baseline or received one during follow up. 525 (7.6%) subjects died during follow up. The subjects on disability pension had a higher mortality rate than the non-retired, the hazards ratio (HR) being 2.78 (95%CI 2.08–3.71) among women and 3.43 (95%CI 2.61–4.51) among men. HR was highest among individuals granted a disability pension at young ages (HR >7), and declined parallel to age at which the disability pension was granted. The higher mortality rate among the retired subjects was not explained by disability pension cause or underlying disease or differences in age, marital status, educational level, smoking habits or drug abuse. There was no significant association between reason for disability pension and cause of death.

**Conclusion:**

Subjects with a disability pension had increased mortality rates as compared with non-retired subjects, only modestly affected by adjustments for psycho-socio-economic factors, underlying disease, etcetera. It is unlikely that these factors were the causes of the unfavorable outcome. Other factors must be at work.

## Background

The majority of subjects granted a disability pension have chronic diseases. The course of these diseases is generally not altered by retirement, in spite of the physical or mental burden of work being relieved [1,2]. In most cases disability pensions are granted because of diseases that are not life threatening, but they may be associated with increased health care utilization [3] and impaired well being [4].

A number of studies have reported markedly higher mortality rates among subjects with disability pensions than among non-retired [5-12]. The cause of the increased mortality rate is unclear. All studies but one [6] speculated that the cause probably was the underlying disease, but there are no published reports where the effects of underlying diseases and other possible confounders, apart from age and sex, are actually taken into account.

We therefore decided to analyze survival among disability pensioners and other subjects in an 18-year follow-up study, including data on disability pension, hospital admissions and mortality data. The aim of the study was to investigate whether disability pensioners have a higher mortality rate than other subjects and, if so, whether it is associated with the underlying disease or diseases or to other potential confounding or effect modifying factors, such as age at retirement, lifestyle factors and socio-economic factors, or whether there is evidence that other factors are involved.

## Methods

### Study population

Data from five on-going population studies in Sweden, with baseline investigations performed between 1980 and 1993, were used for this study. The names of the studies, investigation year, gender, age range, sample sizes, total response rates, and investigation procedures are given in Table 1. Briefly, random samples of the specified age and sex segment of the local general populations were drawn from the national population register of a total of 10,808 subjects, of whom 6,887 (3,738 men and 3,149 women) subjects less than 65 years of age at baseline participated. The response rate was 73% overall and 78% among those less than 65 years of age. In addition, some data were available for all non-participants except for those in the Study of Men Born in 1933, altogether 1,676 subjects (809 women and 867 men).

### Data collection

Postal questionnaires were sent to some of these subpopulations, while others answered questionnaires on location in connection with a medical examination. Data from some of the questionnaires, identical in all five subpopulations, were used for this study. The information obtained included age at the baseline examination, marital status, number of people in the household unit, education, and smoking habits. For this report, marital status was classified as married/cohabiting or not married/cohabiting. Education was classified on a 5-point scale ranging from compulsory school only (= 1) to university education (= 5). Smoking habits were classified as currently smoking or not smoking. In addition, for some of the populations a 5-degree scale was available (never smoked, ex-smoker, currently smoking less than 15 cigarettes per day, 15–24 cigarettes per day or 25 cigarettes per day or more).

In Sweden, the normal old age retirement age is 65 years. The National Social Insurance Board administers all retirement benefits. Information on whether the subjects in the study population had been granted a disability pension at any time from 1971 until 2001 was obtained from the Board. The data obtained included decision date, diagnoses, extent (100%, 75%, 67%, 50% or 25%) and type (temporary or permanent). More than one decision could be taken, for instance first for a temporary and then a permanent disability pension, or first a part time one and then a full time one. The number of decisions ranged from 1 to 6. Data from all decisions were used.

Data on hospital admissions and cause-specific mortality from January 1 of the baseline year until December 31, 2002 were obtained from the National In-patient Registry and the National Cause of Death Registry, respectively. The reasons for disability pension, discharge diagnoses and causes of death were classified according to the ICD versions 8–10 [13], and were 100% complete.

Informed consent on participation in the study was obtained from all participants, oral in the early part of the study and written later on, as required first by the Research Ethics Committees at Uppsala and Göteborg Universities and later by the National Research Ethics Authority. The Committees and the Authority have approved the study on several occasions.

### Statistical analysis

Data were analyzed with the SAS [14] statistical program package. Summary statistics, such as means and measures of dispersion, were computed with traditional parametric methods. Simple differences between the groups regarding continuous data were tested with Student's t-test and nominal or ordinal data with the chi-square test.

January 1 of the examination year or of the year of the postal questionnaire mailing was used as baseline and December 31 2002 was the last day of follow-up. Those who had a disability pension at baseline were regarded as disability pensioners throughout the study. Those who received a disability pension during follow up were regarded as non-retired from baseline until the day before they received their pension, and from then on as disability pensioners. If a new pension decision was taken after the baseline date the observation period of the old decision was terminated the day before the new decision and a new observation period was started on the day of the new decision. For those with more than one decision before baseline, the one in effect at baseline was counted as the first.

Subjects being referents all through the study thus had only one observation period, from baseline to death or end of follow up. Those who were disability pensioners at baseline or became one during follow up had one or more observation periods, the first one starting at baseline and succeeding periods starting when disability pension status changed.

The underlying hazard function and cumulative mortality rate were analyzed with a time dependent Poisson regression procedure adapted to the SAS program package. In these analyses the relation between the hazard function of the various groups was found to be approximately proportional. Hazards ratios (HR) and their 95% confidence intervals (95%CI) were therefore also computed with Cox's proportional hazards regression. Censoring events were termination of a disability pension decision (because of a new one was about to be in effect) or no event until the end of follow up. The outcome variable was death from any cause during follow up. Follow-up time was right truncated at the end of the 18th year because of small numbers.

The effects of potential confounders and effect modifiers on mortality were adjusted for by including these variables as covariates in the model. The results were checked with survival analyses in strata of these variables. P-values less than 5% were regarded as statistically significant.

## Results

### Characteristics of the study population

Of the 6,887 subjects younger than 65 at baseline, 1,683 (24.4%) had a disability pension at baseline or received one during follow up, Table 2. The study population generated a total of 8,669 observation periods and 97,245 person-years of observation. Mean follow-up time per subject was 11.8 years, median 11.0 years and range 0.4–23.0 years. The retired subjects were older than the non-retired subjects, were less often married, and had less education. Men on disability pension were more often smokers and had more often been in hospital for alcohol or drug abuse than others.

The five most common underlying causes of the disability pensions were musculoskeletal disorders (43.3%), psychiatric diseases (19.6%), cardiovascular diseases (9.9%), neurological disorders (6.0%) and trauma (4.5%), Table 3. More women than men had been granted disability retirement status because of musculoskeletal disorders and miscellaneous causes, and more men than women because of cardiovascular diseases, respiratory diseases, endocrine disorders and alcohol or drug abuse.

### Causes of death, mortality rates and time trends

Overall, 525 (7.6%) subjects died during follow-up, Table 4. After adjustment for the influence of age, marital status, educational level and smoking habits, the hazards ratios tended to be greater than unity for most causes of death. The ratios were significantly different from unity for cardiovascular diseases, miscellaneous causes and all causes. For all cause mortality, the hazards ratio was 2.78 (95%CI: 2.08, 3.71) among women and 3.43 (95%CI: 2.61, 4.51) among men. Further adjustment for the influence of the underlying cause of disability pension or hospital admission regardless of cause changed the hazards ratios for all cause mortality only marginally (2.52, 95%CI: 1.88, 3.37 for women and 3.45, 95%CI: 2.47, 4.82 for men).

Hazards ratios in potential confounder strata are shown in Table 5. The hazards ratios were significantly increased whether being responder or non-responder, whether only disability pension status at baseline was taken into account or whether disability pension status was updated during follow up, whether the study population was followed up until 65 years or from 65 years onwards, whether married or not married, whether smoker or non-smoker, and irrespective of retirement cause. The hazards ratio increased in relation to extent of disability pension. Partial disability pension that progressed to full disability pension more than doubled the hazards ratio. Age at first decision had a strong influence on the hazards ratio. Among men the ratio fell from 7.82 among the youngest at the time of the retirement to 2.51 among the oldest. Among women there was a similar trend.

**Figure 1:**
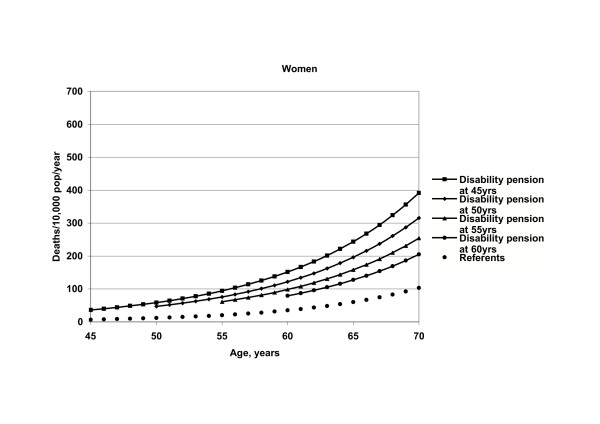
**Hazard function for women**. Hazard function relative to achieved age among non-retired female subjects and disability pensioners according to age at retirement.

**Figure 2:**
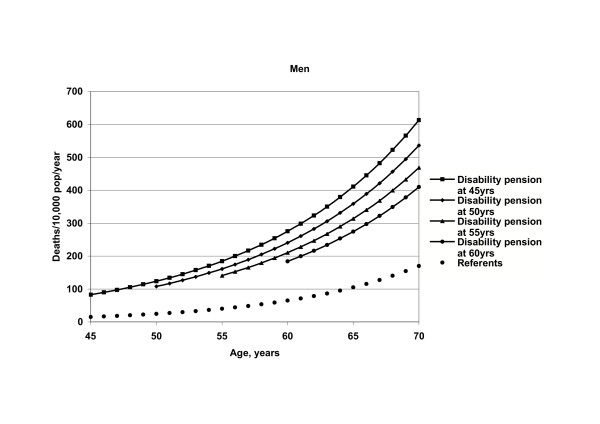
**Hazard function for men**. Hazard function relative to achieved age among non-retired male subjects and disability pensioners according to age at retirement.

In Figures 1 and 2 the inverse relationship between hazards ratio and disability retirement age for women and men is visualized by Poisson regression based hazard function estimates from 45 until 70 years of age. The functional form was the same irrespective of retirement status and retirement age, but the individuals on disability pension had a higher mortality hazard than the non-retired, and those who retired at the youngest age had the most unfavorable hazard. Analyses using follow-up time on the horizontal axis revealed no evidence of initial high mortality among the pensioners.

After 18 years of follow up the cumulative mortality rate adjusted for group differences in age, marital status, education, smoking habits, and hospital admissions for any cause was 5.7% for non-retired women, 7.7% for non-retired men, 19.3% for retired women and 32.7% for retired men. There were no significant differences in mortality pattern between the retired and non-retired subjects.

## Discussion

Men and women who were granted disability pensions had a higher crude mortality rate than non-retired individuals of the same age, sex and place of living, and also after adjustment for a number of potential confounders, including reason for the disability pension and co-morbidity. The hazards ratio for individuals on disability pension as compared with the non-retired was highest among those retiring early in life and then decreased with age at first disability pension decision. There was no evidence of early high mortality among the retired.

The analyses were based on screening data from five population-based cohorts in southern and central Sweden, and on official register data regarding time and cause of disability pension, hospital admissions, and death with little or no data loss. The participation rate at screening was satisfactory and a survival analysis of screening participants and non-responders gave very similar results. We have no data on early retirement other than disability pension, which means that some subjects may have become early non-disability pensioners rather than disability pensioners and were therefore included in the reference group. If these persons had a similar mortality risk as the disability pensioners we have underestimated the mortality difference somewhat. If they were more like other referents the estimates are correct. There was a considerable variation in follow-up time because the various sub-studies were started at different periods of time. However, this circumstance had no differential effect, since the distribution of disability pensioners and referents was similar in the five subpopulations.

The study design causes a certain amount of left truncation of data since those who were disability pensioners at baseline were survivors at least until baseline. The results from analyses with account taken only of the disability pension status at baseline (Table 5) and analyses with the status updated during follow up (Table 4) indicate that the effect of the truncation among women was negligible (OR 2.76 and 2.78, respectively) and a moderate underestimation among men (OR 2.36 and 3.43, respectively. We therefore have no reason to believe that the results are affected by selection or other data bias to such an extent that the conclusions would be affected.

An increased mortality rate among subjects who retire early on disability pension as compared to the non-retired has been found in a number of studies. In an American study of 1,564 men followed for four years, 53% of applicants for disability pension survived as compared to 97% of those not awarded pension [12]. In a Swedish study of 235 men granted a first decision disability pension, a standardized mortality ratio of 2.6 (corresponding to an odds ratio of 4.11) was found [10]. An age adjusted mortality hazards ratio of 7.2 was found among 61 Danish men retired because of illness and 121 referents followed for seven years [5].

In a study of 1,353 Danish men retired because of a disability and 1,353 non-retired men from the same trade union matched by age and geographical area the mortality hazards ratio was 6.8 [9]. In the British Regional Heart Study 7275 men were followed for five years. Compared with those continuously employed at baseline and during follow up, the relative mortality risk ratio for those becoming unemployed or retired due to illness during follow up was 3.14, for those becoming unemployed not due to illness 1.47, and for those becoming retired not due to illness 1.86 after adjustment for geographical area, social class, smoking habits, alcohol consumption, body mass index and pre-existing illness [6]. The effects were not restricted to certain causes of death.

In a previous study based on a small study population (n = 835) we found mortality hazards ratios very similar to those in the present study [15]. In a Danish study of 241,634 men and 254,898 women, followed from 1986 to 1996, the standardized mortality ratio in employed subjects was 0.59/0.51 for men/women, for disability benefit recipients 2.31/1.66, for the early retired 0.88/0.72, and for other occupationally inactive individuals 0.84/0.67 [11]. An approximate mortality relative risk estimate for retired subjects versus non-retired subjects based on recalculation of their data would be 3.20 for men and 2.63 for women, close to our estimates. Tsai et al. found that subjects retiring before age 65 had higher mortality rates than those retiring at 65 years [16].

All cited studies thus reported the same main finding as in the present study, i.e., subjects retiring due to disability have a poorer survival rate than the non-retired. In most studies, including ours, there was no initial high mortality rate among the retired. The main advantages of the present study are the inclusion of both men and women, that the data set was based on screening as well as register data, providing highly valid exposure and outcome data, the possibility of adjustment for a wide range of potential confounders and other effect modifiers, and the size of the study population, larger than in most previous studies, providing great precision in most of the estimates. We appear to be the first to account for confounding due to underlying diseases.

There are a number of possible explanations for the higher mortality rate among the disability pensioned men and women. First, the retired subjects were all diagnosed with a disease, although in the vast majority with diseases that are normally non-fatal. When we analyzed the data per disability pension diagnosis group, the subjects with "benign" retirement diagnoses, such as musculoskeletal disease, also had increased mortality rates. Further evidence against a mere effect of the underlying disease are the lack of correspondence between retirement diagnoses and causes of death, and the fact that the increased mortality risk persisted when retirement and hospital discharge diagnoses were taken into account. It is therefore unlikely that the differences in mortality were entirely caused by known underlying diseases.

A second possible explanation might be that the retired subjects had other severe disease conditions than those indicated by the disability pension diagnoses. This alternative is supported by the lack of correspondence between retirement diagnosis and cause of death in the present study, and the high long-term health care utilization rate after disability pension, with no significant correlation between hospital discharge diagnoses and disability pension diagnoses as previously reported from a subset of this study population [3]. However, the adjustments for the influence of hospital admission diagnoses that we made should have taken the severity factor into account.

A third possible explanation might be that factors other than the disease per se, such as an unfavorable lifestyle or psycho-socio-economic factor profile, contributed to the increased mortality rate. It has been shown in several studies [6,17,18], as well as in the present one, that subjects retiring due to illness have an unfavorable risk factor profile as compared with others (less education, smoke more, drink more and are more often single). There is evidence that an unfavorable psycho-socio-economic situation increases the risk of health deterioration and vice versa [19]. However, adjustments for such factors had only marginal effects on the hazards ratio.

A fourth possible explanation might be damage caused by the disability pension process per se. Most retirements due to disability are involuntary as opposed to normal old age retirement. This means that disability pension may per se contain a damaging factor in addition to the underlying disease. In Western societies, work and self-sufficiency have high status [20]. A substantial part of the social network, the work-related part, is lost with disability pension, which may be a negative health factor. This means that the job loss associated with disability pension might mean loss of one's identity and position in society, a sort of bereavement [21]. In this situation, the identity of being a sick person might replace the identity of being a working and self-sufficient one. There is evidence that this type of changed identity affects well being [22-24]. This view is also supported by many studies reporting that job loss is associated with decreased survival rates [25-30], even among apparently healthy subjects [6].

If the disability retirement per se includes a damaging factor one might wonder whether the outcome would have been different if the retired person had not been granted a disability pension but were allowed to stay at work with some kind of adjusted work situation. This is an important scientific and medical issue that warrants further research. The final solution to the problem might be a randomized clinical trial, even though such a design involves ethical and other controversial issues.

## Conclusion

In conclusion, we found that men and women with a disability pension had increased mortality rates as compared with the non-retired, and that these were only modestly affected by adjustments for psycho-socio-economic factors, underlying disease, and other factors. There was no clear association between retirement diagnosis and cause of death, indicating that the underlying disease or obvious confounding is unlikely to be the cause of the unfavorable outcome. Most probably other deleterious factors are at work.

## Competing interests

The author(s) declare that they have no competing interests.

## Authors' contributions

All authors designed the study and collected the data in their subpopulation. TW and KS obtained and edited additional register data. TW, HW and KS performed the analyses and drafted the manuscript. All authors participated in the discussions of the draft outline and contributed later with text revisions, table revisions and figure revisions. All authors have seen and approved the final version of the manuscript.

## Pre-publication history

The pre-publication history for this paper can be accessed here:



## Figures and Tables

**Table 1:** Study subpopulations. Data source, examination year, gender, age range at baseline, sample size, response rate and investigation procedure for the five subpopulations

Subpopulation	Investigation		Gender	Age range	Sample size	Responders	Response rate, %	< 65 years at baseline	Investigation procedure
								
	Year	Place							
BEDA	1980	Gothenburg	women	38–64	1704	1413	82.9	1413	Questionnaires + medical examination
Men Born in 1933	1983	Gothenburg	men	50	1016	776	76.4	776	Questionnaires + medical examination
ESKIL	1986	Eskilstuna	men	30–54	625	461	73.8	461	Postal questionnaire
Men Born in 1943	1993	Gothenburg	men	50	1463	798	54.5	798	Questionnaires + medical examination
Public Health Cohort Uppsala	1993	Uppsala	women	25-	2999	2249	75.0	1736	Postal questionnaire
Public Health Cohort Uppsala	1993	Uppsala	men	25-	3001	2155	71.8	1703	Postal questionnaire

Total					10808	7852	72.6	6887	

**Table 2:** Characteristics of the study population. Characteristics of women and men with a disability pension at baseline or during follow up, and non-retired women and men

	Disability pension				Non-retired					
				
	Women		Men		Women		Men		p for difference	
				
	Mean or %	SD	mean or %	SD	mean or %	SD	mean or %	SD	pension status	gender
No of subjects	892		791		2257		2947			
No. of observation periods	1887^1)^		1578^2)^		2257		2947			
No. of person-years	9479		6923		35528		40320			
Age at baseline	53.8	8.09	52.5	8.55	44.7	10.06	44.6	8.65	<0.0001	n.s.
Married, %	73.8		63.4		78.6		80.4		<0.001	n.s.
Mandatory education only	70.8		55.4		43.0		31.0		<0.0001	<0.0001
Household size	2.3	1.19	2.4	1.49	2.9	1.22	2.9	1.32	<0.0001	n.s.
Smokers, %	37.8		42.2		33.3		30.1		<0.005	<0.01
Hospital care for alcohol or drug abuse, %	0		5.4		0.4		0.9		<0.01	<0.005

^1) ^634 observation periods before and 1258 periods after disability pension.

^2) ^592 observation periods before and 992 periods after disability pension.

**Table 3:** Reason for disability pension. Underlying diagnosis for those who were on disability pension at baseline or received one during follow up

	Women		Men		
			
Cause of disability pension	n	%	n	%	p
Musculoskeletal disease	427	47.9	309	39.1	<0.0005
Psychiatric disease	160	17.9	169	21.4	n.s.
Alcohol or drug abuse	3	0.3	33	4.2	<0.0001
Cardiovascular disease	63	7.1	104	13.2	<0.0001
Myocardial infarction	22	2.5	56	7.1	<0.0001
Stroke	9	1.0	16	2.0	n.s.
Other cardiovascular disease	32	3.6	32	4.1	n.s.
Neurological disease	53	5.9	48	6.1	n.s.
Trauma	33	3.7	43	5.4	n.s.
Respiratory disease	21	2.4	33	4.2	<0.05
Tumor	32	3.6	19	2.4	n.s.
Endocrine disorder	13	1.5	25	3.2	<0.05
Gastrointestinal disease	14	1.6	9	1.1	n.s.
Dermatologic disease	10	1.1	6	0.8	n.s.
Infectious disease	4	0.5	9	1.1	n.s.
Urinary tract disease	4	0.5	3	0.4	n.s.
Miscellaneous	58	6.5	14	1.8	<0.001

All causes	892	100	791	100	

**Table 4:** Causes of death. Underlying causes of death during 18 years of follow up among disability pensioners and referents

	Women						Men					
		
	Disability pension		Non-retired				Disability pension		Non-retired			
								
	n^1)^	rate^2)^	n^1)^	rate^2)^	HR^3)^	95%CI^4)^	n^1)^	Rate^2)^	n^1)^	rate^2)^	HR^3)^	95%CI^4)^
Infectious disease	2	22	0	0	4.46	0.32, 61.98	0	0	0	0		
Tumors	47	527	56	248	1.92	1.25, 2.95	31	392	44	149	1.72	0.997, 2.95
Endocrine disease	1	11	2	9	0.89	0.07, 10.89	4	51	2	7	17.58	2.17, 142.36
Psychiatric disease	5	56	2	9	5.53	0.95, 32.16	2	25	5	17	1.32	0.20, 8.69
Alcohol or drug abuse	2	22	1	4	18.14	0.72, 459.46	2	25	5	17	1.32	0.20, 8.69
Neurological disease	3	34	2	9	2.42	0.36, 16.27	4	51	0	0		
Cardiovascular disease	36	404	17	75	3.91	2.11, 7.24	49	619	44	149	3.34	2.05, 5.47
Myocardial infarction	15	168	8	35	3.64	1.45, 9.15	36	455	35	119	3.03	1.73, 5.30
Stroke	10	112	2	9	7.24	1.54, 33.93	4	51	3	10	4.32	0.70, 26.67
Other cardiovascular disease	11	123	7	31	3.21	1.14, 9.02	9	114	6	20	4.80	1.38, 16.66
Respiratory disease	9	101	7	31	2.69	0.92, 7.88	9	114	3	10	8.96	1.94, 41.44
Gastrointestinal disease	5	56	3	13	3.14	0.66, 14.89	6	76	1	3	26.00	2.34, 288.45
Trauma	5	56	4	18	4.89	1.01, 23.57	7	88	20	68	1.74	0.65, 4.68
Miscellaneous causes	15	168	6	27	2.64	1.49, 4.70	42	531	25	85	4.76	2.78, 8.13

All causes	128	1435	99	439	2.78	2.08, 3.71	154	1947	144	489	3.43	2.61, 4.51

^1) ^number of deaths, ^2) ^number of deaths per 10,000 population, ^3) ^age adjusted hazards ratio, ^4) ^95% confidence interval.

**Table 5:** Hazards ratios in strata of potential confounders. Age adjusted mortality hazards ratios (HR) and 95% confidence intervals (95%CI) among women and men on disability pension versus their referents in strata of potential confounders and other effect modifying variables

	Women			Men		
		
	Fatalities^1)^.	HR	95%CI	Fatalities^1)^.	HR	95%CI
Non-responders	51/38	2.69	1.69–4.29	16/26	2.82	1.35–5.88
Retirement status at baseline only	58/169	2.76	2.04–3.74	51/247	2.36	1.73–3.22
Follow-up until 65 yrs	53/50	3.49	2.23–5.48	98/108	4.94	3.58–6.81
Follow-up from 65 yrs	88/86	2.57	1.86–3.55	57/44	2.13	1.36–3.36
Extent of disability pension						
25–50%	8/99	1.63	0.78–3.40	12/144	2.23	1.20–4.16
67–75%	3/99	2.86	0.91–9.06	2/144	2.53	0.61–10.44
100%	117/99	3.01	2.24–4.05	140/144	3.83	2.90–5.06
Increase of extent	94/99	2.60	1.89–3.58	118/144	3.45	2.54–4.68
Married	90/71	2.77	1.97–3.91	62/83	2.87	1.93–4.26
Not married	38/28	2.86	1.69–4.82	46/20	6.54	3.62–11.81
Compulsory education only	91/65	2.32	1.65–3.26	71/35	5.24	3.28–8.37
More than compulsory education.	35/34	4.02	2.34–6.90	37/67	3.10	1.92–5.00
Smokers	62/47	2.26	1.48–3.43	100/61	3.36	2.30–4.93
Non-smokers	66/52	2.96	1.99–4.39	52/80	2.79	1.84–4.25
Reason for disability pension						
Musculoskeletal	40/99	1.74	1.18–2.57	42/144	2.08	1.41–3.09
Psychiatric	28/99	3.53	2.28–5.46	35/144	3.76	2.55–5.54
Cardiovascular	15/99	3.78	2.16–6.62	22/144	3.65	2.22–6.00
Neurological	6/99	2.34	1.02–5.37	10/144	3.81	1.94–7.46
All other causes	41/99	3.60	2.42–5.37	45/144	5.75	3.89–8.52
Age at first disability pension decision						
16–34	1/99	3.60	0.49–26.27	3/144	7.82	2.44–25.05
35–44	18/99	8.14	4.85–13.67	17/144	4.81	2.91–7.96
45–54	33/99	2.71	1.82–4.03	62/144	4.17	3.05–5.70
55–64	76/99	2.21	1.56–3.12	72/144	2.51	1.71–3.68

^1) ^Number of fatalities among retired and non-retired subjects
